# Semaphorin 3A Increases FAK Phosphorylation at Focal Adhesions to Modulate MDA-MB-231 Cell Migration and Spreading on Different Substratum Concentrations

**DOI:** 10.1155/2017/9619734

**Published:** 2017-01-15

**Authors:** Scott Gehler, Frances V. Compere, Alex M. Miller

**Affiliations:** Biology Department, Augustana College, Rock Island, IL 61201, USA

## Abstract

Interactions between integrin-mediated adhesions and the extracellular matrix (ECM) are important regulators of cell migration and spreading. However, mechanisms by which extracellular ligands regulate cell migration and spreading in response to changes in substratum concentration are not well understood. Semaphorin 3A (Sema3A) has been shown to inhibit cell motility and alter integrin signaling in various cell types. We propose that Sema3A alters focal adhesions to modulate breast carcinoma cell migration and spreading on substrata coated with different concentrations of ECM. We demonstrate that Sema3A inhibits MDA-MB-231 cell migration and spreading on substrata coated with high concentrations of collagen and fibronectin but enhances migration and spreading at lower concentrations of collagen and fibronectin. Sema3A increases focal adhesion kinase phosphorylation at tyrosine 397 (pFAK^397^) at focal adhesions on all substratum concentrations of collagen and fibronectin but decreased pFAK^397^ levels on laminin. Rho-associated protein kinase (ROCK) inhibition blocks the Sema3A-mediated effects on cell migration, spreading, and pFAK^397^ at focal adhesions when cultured on all concentrations of collagen. These results suggest that Sema3A shifts the optimal level of cell-matrix adhesions to a nonoptimal ECM coating concentration, in particular collagen, to yield maximal cell migration and spreading that may be mediated through a ROCK-dependent mechanism.

## 1. Introduction

Cell migration is critical to normal and pathological processes, including embryogenesis, wound healing, angiogenesis, and tumor metastasis [1]. Cell migration involves a series of coordinated processes that include leading edge protrusion, attachment of the leading edge to the stromal environment through cell-substratum attachments, and retraction of the trailing edge of the cell by reducing cell-substratum attachments in order to promote translocation [2]. Integrins are transmembrane receptors that form cell-substratum adhesions by linking the ECM to the underlying cytoskeleton through various scaffolding proteins [3]. Integrins sense both chemical and mechanical properties pertaining to the extracellular environment that alter signal transduction pathways to influence various cellular responses, such as cell shape, migration, proliferation, and gene transcription [4]. Although the role of integrin signaling in regulating various cellular responses has been extensively studied, it is not well understood how integrin-cytoskeletal linkages are altered by changes by the different properties of the ECM.

Studies have shown that cells exhibit a biphasic relationship between cell migration speed and increasing adhesion strength, suggesting that cells experience an optimal level of cell-substratum adhesion strength to facilitate maximal cell migration [5, 6]. For instance, if the strength of cell-substratum adhesions is too low, cells do not exhibit sufficient traction to effectively move, resulting in reduced motility. However, if the strength of cell-substratum adhesions is too high, cell-substratum attachments are too strong for the cell to overcome, resulting in reduced motility. Various studies have produced results that support the relationship between adhesion strength and cell motility by modulating substrate concentration, integrin expression, or integrin-ECM binding affinity [5–8]. In addition, other studies have demonstrated a biphasic relationship between adhesion strength and cell spreading [9, 10], while changes in substratum concentration have been shown to alter cell morphology [11]. These findings suggest that adhesion strength modulates various cellular responses that are dependent on changes to the actin cytoskeleton. While the biphasic relationship between adhesion strength and cell shape, motility, and spreading is well supported, how extracellular ligands modulate changes in cell-substratum adhesiveness to alter cellular responses is not well understood.

Semaphorins are factors that were originally characterized for their role in axon pathfinding during neural development [12]. However, semaphorins play important roles in other physiological processes, such as participating in the immune response, angiogenesis, and cancer [13]. For instance, semaphorins have been shown to inhibit tumor progression and metastasis of various types of cancer [12]. In particular, semaphorin 3A (Sema3A) displays tumor suppressing effects on prostate and breast carcinoma cell migration and invasion in vitro [14, 15]. Furthermore, overexpression of Sema3A suppresses tumor growth and metastasis in vivo using xenograft mouse models [16–18]. Inhibition of the Sema3A coreceptor, neuropilin-1, blocks the Sema3A-mediated effects on tumor metastasis [18]. Indeed, reduced expression of Sema3A is correlated with breast carcinoma and melanoma progression in humans [17, 19]. Collectively, these findings suggest that Sema3A plays a pathophysiological role in tumor progression.

Sema3A increases integrin expression and cell adhesion in breast epithelial carcinoma cells while treatment with an *α*2*β*1 antibody blocks the Sema3A-induced increases on cell adhesion [20]. Semaphorins regulate integrin function and target cytoskeletal changes through various signaling mechanisms [12]. For instance, Sema3A stimulates the growth of hippocampal dendrites through integrin-dependent phosphorylation of focal adhesion kinase (FAK) [21]. Ablation of FAK blocks Sema3A-induced assembly of integrin-mediated adhesions and axonal remodeling of hippocampal neurons [22]. Semaphorin 7A enhances axon outgrowth through integrin-dependent activation of MAPK [23]. Toyofuku et al. [24] showed that Sema3A inhibits neurite outgrowth of dorsal root ganglion neurons through downregulation in integrin-dependent cell adhesions. These studies implicate an important role for integrin-dependent signaling in mediating semaphorin-induced cellular responses in neuronal and carcinoma cells.

Maximal cell motility and spreading is regulated by cell-substratum adhesive interactions that are dependent on integrin expression, integrin-ECM binding affinity, and the concentration of the substrate. However, it is not clear whether different ECM molecules or changes in substratum concentration can modulate the Sema3A-mediated changes in cell motility and spreading. Therefore, we assessed whether Sema3A alters the motility and spreading of MDA-MB-231 breast carcinoma cells on substrata coated with different concentrations of ECM through changes in cell-substratum adhesion dynamics. If Sema3A increases focal adhesions in MDA-MB-231 cells, then we predict that Sema3A treatment would inhibit cell motility and spreading on substrata coated with high concentrations of ECM. However, on nonoptimal substratum concentrations, we would expect enhanced cell motility and spreading through increased focal adhesions. Our findings demonstrate that Sema3A inhibits cell motility and spreading on substrata coated with high concentrations of collagen or fibronectin but enhances motility and spreading when collagen or fibronectin coating concentration is reduced. In addition, Sema3A enhances pFAK^397^ levels at focal adhesions on all concentrations of collagen and fibronectin but reduces pFAK^397^ levels at focal adhesions on laminin, suggesting that Sema3A modulates focal adhesions in response to altered substrate concentration. Inhibition of Rho-associated kinase (ROCK) blocked the Sema3A-mediated changes in cell motility, spreading, and pFAK^397^ at focal adhesions on collagen. These findings suggest that Sema3A tunes the cellular responses to nonoptimal ECM coating concentrations through increased focal adhesions that may involve a ROCK-dependent mechanism. These observations have potential therapeutic considerations for tumor metastasis in light of the changes in integrin expression, ECM deposition, and Sema3A expression during tumor progression.

## 2. Materials and Methods

### 2.1. Reagents

Rat tail collagen type I (#354236) and human fibronectin (#354008) were obtained from Corning. Mouse laminin I (#3400-010-01) was purchased from Trevigen, Inc. Recombinant human IgG1 Fc (#110-HG) and recombinant human semaphorin 3A Fc chimera (#1250-S3) were purchased from R&D Systems. A ROCK inhibitor (Y-27632; #688000) was purchased from EMD Millipore Corporation. Mouse anti-human FAK (pY397; #611806) was purchased from BD Transduction Laboratories. Phalloidin–Tetramethylrhodamine B isothiocyanate (#P1951) was obtained from Sigma-Aldrich. Alexa488 goat anti-mouse IgG (#115-545-003) was from Jackson ImmunoResearch Laboratories, Inc. CellTiter 96® AQueus One Solution (#G3580) was purchased from Promega Corporation. Characterized fetal bovine serum (#SH30071.03) and penicillin-streptomycin (#SV30010) were obtained from HyClone. All culture media was from Corning.

### 2.2. Cell Lines and Cell Culture

MDA-MB-231 breast carcinoma cells were generously provided by Dr. Patricia J. Keely (University of Wisconsin-Madison). Cells were cultured in DMEM containing 10% FBS plus penicillin-streptomycin. For all experiments, “low” coating concentration was 1–3 *μ*g/mL, “intermediate” coating concentration was 10 *μ*g/mL, and “high” coating concentration was 50–100 *μ*g/mL of collagen, fibronectin, or laminin.

### 2.3. Scratch Assays

16 hours prior to cell seeding, wells of a 12-well tissue culture plate were coated with different concentrations of collagen, fibronectin, or laminin. All wells were blocked with 10 mg/mL fatty acid-free bovine serum albumin in PBS for 1 hour at room temperature prior to cell plating. 800 *μ*L of MDA-MB-231 cells (250,000 cells/mL) suspended in complete media (containing 10% FBS) was plated into each well. After 24 hours, cells were rinsed and serum-starved for 18 hours prior to scratch formation. Following the production of a wound using a 200 *μ*L pipet tip, detached cells were rinsed and fresh serum-free media containing either 100 ng/mL IgG1 Fc or Sema3A were added to the cells and were permitted to migrate for 18 hours at 37°C/5% CO_2_. Images of the wounds were obtained using a 10x objective on an Olympus IX-51 inverted microscope equipped with a QImaging CCD camera. Images were acquired using QImaging Q-Capture Pro. Cell migration (% area closure) was quantified by measuring the area of the wound immediately following wound formation and after 24 hours using ImageJ analysis software (https://imagej.nih.gov/ij).

### 2.4. Time-Lapse Video Microscopy

A 35 mm tissue-culture dish was coated with different concentrations of collagen, fibronectin, or laminin. Prior to cell seeding, the ECM was removed and dishes were blocked with 10 mg/mL fatty acid-free BSA in PBS for 30 minutes at room temperature. Following block, cells were resuspended in serum-free DMEM containing antibiotics and 2 mL of cell suspension (25,000 cells/mL) was added to the dish and permitted to attach for 16 hours at 37°C/5% CO_2_. The next day, growth media was removed from each dish and replaced with fresh serum-free media containing 50 mM HEPES. 30 minutes prior to imaging, 100 ng/mL IgG1 Fc or Sema3A was added to the cells and the culture dish was placed on a heated microscope stage. Phase-contrast images were captured using a 10x objective on an Olympus IX-51 inverted microscope equipped with a QImaging QIClick cooled-CCD camera. Images were captured every 1 minute for 60 minutes using QImaging Q-Capture Pro. Cell speed and directional persistence were quantified using MTrackJ (https://www.imagescience.org).

### 2.5. Cell Spreading Assays

16 hours prior to cell seeding, 22 × 22 mm glass coverslips were coated with 200 uL of different concentrations of collagen, fibronectin, or laminin. All coverslips were blocked with 200 *μ*L of 10 mg/mL fatty-acid-free bovine serum albumin for 30 minutes at room temperature prior to cell plating. 200 *μ*L of cells (500,000 cells/mL) containing 100 ng/mL IgG1 Fc or Sema3A in serum-free media was added to each coverslip. Cells were permitted to attach and spread for 30 minutes at 37°C/5% CO_2_. After 30 minutes, cells were fixed with 200 uL of 0.25% glutaraldehyde for 10 minutes. All coverslips were washed with PBS two times prior to the addition of 200 *μ*L of 0.5 *μ*M TRITC-phalloidin containing 0.5% Trition X-100. The coverslips were incubated for 45 minutes at room temperature and then rinsed and mounted with ProLong Antifade (Molecular Probes). Images were captured using a 40x objective on a Zeiss Axiovert upright fluorescent microscope equipped with a AxioCam MRm camera. Cell area was quantified using ImageJ.

### 2.6. Immunofluorescence

MDA-MB-231 cells were plated onto 22 × 22 mm coverslips coated with different concentrations of collagen, fibronectin, or laminin. Cells were detached and pretreated with 100 ng/mL Fc control or Sema3A and 10 *μ*M Y-27632 (or control), when applicable, for 30 minutes prior to plating onto the coverslips. After 1-hour incubation at 37°C/5% CO_2_, cells were fixed with cold 4% paraformaldehyde for 15 minutes. Cells were rinsed with PBS and quenched with 0.15 M glycine for 10 minutes at room temperature. Following rinse, 0.1% Triton X-100 (in PBS) was added to the cells for 10 minutes at room temperature. Cells were blocked with 10% normal goat serum (Jackson ImmunoResearch Laboratories, Inc.) for 1 hour at room temperature. Cells were incubated in a humidified chamber overnight at 4°C with 1 : 100 mouse anti-human FAK (pY397) antibody in 10% normal donkey serum. After rinsing, cells were incubated with 1 : 800 Alexa488 goat anti-mouse IgG plus 0.5 *μ*M TRITC-phalloidin (Sigma-Aldrich) for 1 hour at room temperature. Following extensive rinsing, coverslips were mounted with ProLong Antifade. Images were analyzed using a 100x objective on a Zeiss Axiovert upright fluorescence microscope equipped with a AxioCam MRm camera, and images were acquired using AxioVision 4.7 software.

### 2.7. Quantification of pFAK^397^ Staining

Focal adhesions were quantified using two methods. The average total surface area occupied by focal adhesions was quantified as described previously [25]. Briefly, images underwent binarization and thresholding of pFAK^397^ staining. Total surface area occupied by pFAK^397^ for each cell was quantified using the analyze particles function of ImageJ. The average total surface area occupied by pFAK^397^ for each cell was normalized to the total cell area as determined by TRITC-phalloidin. This approach accounted for variations in the average cell area as a result of different treatment conditions.

### 2.8. Proliferation Assays

16 hours prior to cell seeding, wells of a 96-well plate were coated with different concentrations of collagen, fibronectin, or laminin. All wells were blocked with 10 mg/mL fatty acid-free bovine serum albumin in PBS for 30 minutes at room temperature prior to cell plating. 100 *μ*L of cells (50,000 cells/mL) suspended in complete media (containing 10% FBS) was plated into each well and incubated at 37°C/5% CO_2_. After 24 hours, the complete media were removed and replaced with 100 *μ*L of serum-free media containing 100 ng/mL IgG1 Fc or Sema3A. Cells were incubated for 24 hours at 37°C/5%/CO_2_. After 24 hours, 20 *μ*L of CellTiter 96 AQ_ueous_ One Solution Reagent was added to each well and the plate was incubated at 37°C/5% CO_2_ for 1 hour. Cell proliferation for each condition was determined by measuring the absorbance at 490 nm using a VersaMax microplate reader (Molecular Devices).

### 2.9. Cell Shape Analysis

Circularity and aspect ratio were used to measure cell shape as previously described [26]. Both cell circularity and aspect ratio were used to measure the roundness of a cell. Cell images captured from the cell spreading assay were analyzed for circularity and aspect ratio. ImageJ was used to measure the area and perimeter of each cell and circularity was determined by (4*π*  ×  area/perimeter^2^). Aspect ratio describes the elongation of a cell by dividing the length of the major axis by the length of the minor axis of a cell (major axis/minor axis) as measured using ImageJ.

### 2.10. Production of Digital Images

Digital images were processed and produced using Adobe Photoshop CS5 (Adobe Systems, San Jose, CA).

## 3. Results

### 3.1. Sema3A Inhibits MDA-MB-231 Cell Motility at High Substratum Concentrations but Enhances Motility at Lower Substratum Concentrations

**Figure 1:**
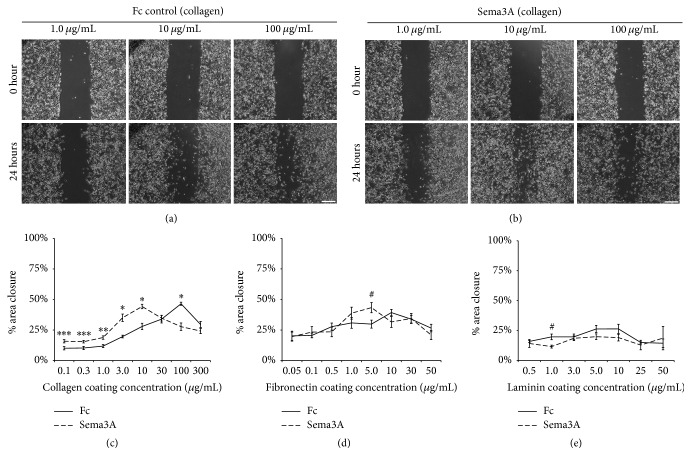
Sema3A enhances cell migration at low coating concentrations of collagen but inhibits migration at high coating concentrations of collagen. ((a) and (b)) Representative images of MDA-MB-231 cells cultured on substrata coated with low (1.0 *μ*g/mL), intermediate (10 *μ*g/mL), and high (100 *μ*g/mL) concentrations of collagen in the presence of 100 *μ*g/mL IgG1 Fc control or Sema3A. Scale bar = 100 *μ*m. ((c)–(e)) Cell migration as measured by average % area closure from cells cultured on different coating concentrations of collagen (c), fibronectin (d), or laminin (e) in the presence of IgG1 Fc control or Sema3A. Sema3A enhanced migration at low and intermediate coating concentrations of collagen by 59% and 58%, respectively, but inhibited migration by 40% at high coating concentrations of collagen. However, although Sema3A enhanced migration by 45% at low coating concentrations of fibronectin, there was no statistically significant reduction in migration at high coating concentrations of fibronectin. Overall, Sema3A did not have any significant effect on laminin. Data are presented as mean% area closure ± SEM from a minimum of three independent experiments. ^*∗*^*p* < 0.001, ^*∗∗*^*p* < 0.005, ^*∗∗∗*^*p* < 0.01, and ^#^*p* < 0.05 indicate statistical significance relative to IgG1 Fc control; two-sample *t*-test.

Studies have shown that maximal cell speed is regulated by cell-substratum adhesion strength that is influenced by substratum concentration [5, 6]. Sema3A has been found to increase integrin receptor expression and cell adhesion in breast cancer cells and inhibit breast cancer cell motility [15, 20]. However, the means by which extracellular cues, such as Sema3A, modulate changes in cell-substratum adhesion strength to alter cancer cell migration speed are not clearly elucidated. Therefore, we determined whether Sema3A alters the motility response of MDA-MB-231 breast carcinoma cells on increasing coating concentrations of type I collagen, fibronectin, and laminin I. MDA-MB-231 carcinoma cells express the Sema3A coreceptors, plexin A1 and neuropilin-1, making it a suitable cell line for this study [16]. Cell motility was assessed using a scratch assay by culturing MDA-MB-231 breast carcinoma cells on a substratum coated with increasing concentrations of collagen in the presence of either IgG1 Fc control or Sema3A (Figures 1(a) and 1(b)). In the IgG1 Fc control condition, MDA-MB-231 cell migration exhibited a biphasic response to increasing coating concentrations of collagen and fibronectin, which is consistent with previously published results (Figures 1(c) and 1(d)) [5, 6]. On substrata coated with lower concentrations of collagen (0.1–10 *μ*g/mL), Sema3A enhanced area closure by 50–78% over IgG1 Fc control (Figure 1(c)). However, on substrata coated with high concentrations of collagen (100 *μ*g/mL), Sema3A inhibited motility by 40%. The inhibitory effect of Sema3A at high collagen coating concentrations is consistent with previous studies [15, 20]. However, the observation that Sema3A enhanced the motility response at lower concentrations of collagen has not been documented to our knowledge. Interestingly, Sema3A did not have an inhibitory effect on cells cultured on fibronectin but enhanced motility by 45% on substrata coated with 5 *μ*g/mL fibronectin compared to IgG1 Fc control (Figure 1(d)). Overall, Sema3A did not have any significant effect on MDA-MB-231 cell migration when plated on laminin (Figure 1(e)). The effects of Sema3A on all substratum concentrations were not a consequence of changes in cell proliferation (Supplemental Figure 1 in Supplementary Material available online at https://doi.org/10.1155/2017/9619734).

**Figure 2:**
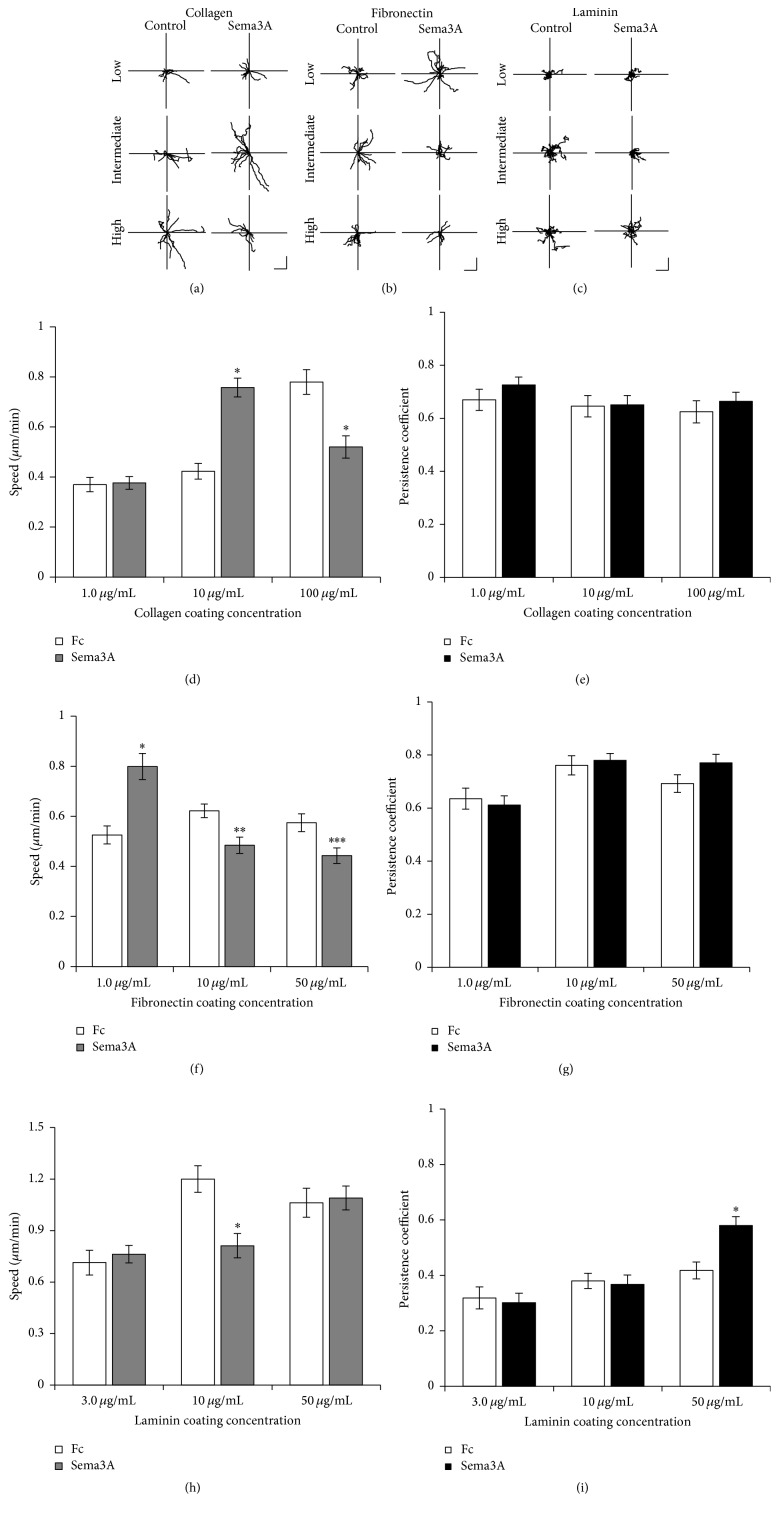
Sema3A regulates cell speed, but not persistence, to alter motility dynamics on collagen and fibronectin, but not laminin. ((a)–(c)) Rose plots showing the migration trajectories of 10 representative tracks for collagen (a), fibronectin (b), and laminin (c) on substrata coated with low, intermediate, and high concentrations of ECM. Scale bar = 10 *μ*m. Average cell speed and directional persistence were measured for collagen ((d) and (e)), fibronectin ((f) and (g)), and laminin ((h) and (i)). Sema3A enhanced motility at lower coating concentrations of both collagen and fibronectin but inhibited motility at higher coating concentrations of collagen and fibronectin. Interestingly, Sema3A inhibited motility on at intermediate coating concentrations of laminin. Graphed data represent the mean ± SEM from a minimum of 30 cells from four independent experiments. ^*∗*^*p* < 0.001, ^*∗∗*^*p* < 0.005, and ^*∗∗∗*^*p* < 0.01 indicate statistical difference relative to IgG1 Fc control; two-sample *t*-test.

To determine whether Sema3A has distinct effects on the motility dynamics of MDA-MB-231 cells plated on collagen, fibronectin, and laminin, we performed time-lapse video microscopy. In general, the migration paths of cells cultured on collagen and fibronectin were more persistent than those cultured on laminin (Figures 2(a)–2(c)). Sema3A-treated cells plated on substrata coated with intermediate concentrations of collagen exhibited enhanced migration speed approximately 2-fold over IgG1 Fc control, while cells on substrata coated with high concentrations of collagen experienced ~33% reduction in cell speed after treatment with Sema3A (Figure 2(d)). Relative to IgG1 Fc control, directional persistence of cells plated on collagen did not change following treatment with Sema3A (Figure 2(e)). Sema3A enhanced cell speed by ~52% on substrata coated with low concentrations of fibronectin but inhibited cell speed at intermediate (~22%) and high coating concentrations (~23%) of fibronectin compared to IgG1 Fc control (Figure 2(f)). Directional persistence was similar between control and Sema3A treatment on all concentrations of fibronectin (Figure 2(g)). These observations for both collagen and fibronectin are consistent with the trends observed from the scratch assay (Figure 1). While the scratch assay produced similar trends as the time-lapse results, the data were not always statistically significant. This could be due to contributions of cell-cell interactions in the scratch assay versus single cell analysis of time-lapse video microscopy. Consistent with the results presented in Figure 1, Sema3A had no effect on cell speed on substrata coated with low and high concentrations of laminin but inhibited cell speed at intermediate coating concentrations of laminin (Figure 2(h)). Interestingly, Sema3A increased directional persistence on substrata coated with high concentrations of laminin but had no effect on substrata coated with lower laminin concentrations (Figure 2(i)). The lack of any observed effect of Sema3A at intermediate coating concentrations of laminin in the scratch assay may have been due to the low directional persistence of the cell trajectories. Collectively, these results suggest that Sema3A shifts the motility curve, in particular for collagen and fibronectin, such that, at low and intermediate substrate concentrations, Sema3A enhances MDA-MB-231 cell motility, whereas, at higher substrate concentrations, Sema3A has inhibitory effects on cell motility.

### 3.2. Sema3A Inhibits MDA-MB-231 Cell Spreading at High Substratum Concentrations but Enhances Spreading at Lower Substratum Concentrations

**Figure 3:**
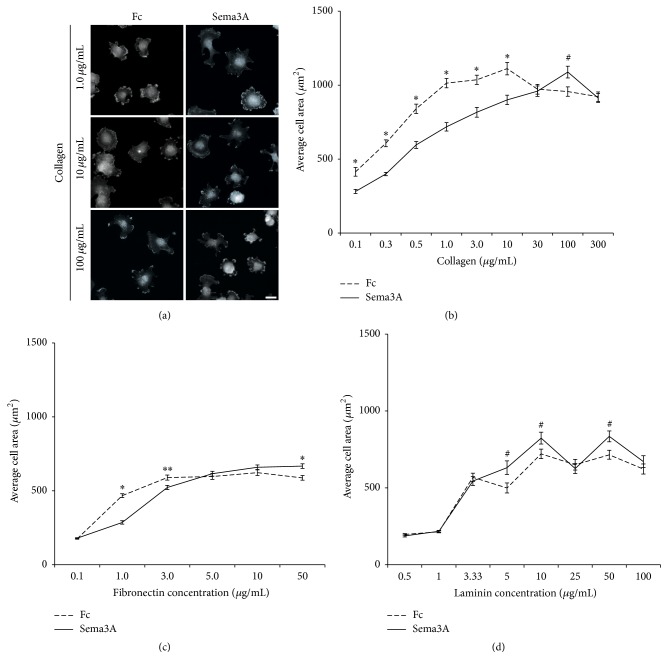
Sema3A increases cell spreading on low and intermediate coating concentrations of collagen and fibronectin but inhibits spreading at high coating concentrations of collagen and fibronectin. (a) Representative images of cells attached to substrata coated with low (1.0 *μ*g/mL), intermediate (10 *μ*g/mL), and high (100 *μ*g/mL) concentrations of collagen that were stained using TRITC-phalloidin. Scale bar = 20 *μ*m. Average area of cells exposed to increasing substratum concentrations of collagen (b), fibronectin (c), and laminin (d). Sema3A enhanced cell spreading at lower coating concentrations of collagen and fibronectin but inhibited spreading at high coating concentrations of collagen and fibronectin. Sema3A inhibited spreading at various coating concentrations of laminin but did not have any stimulatory effect on cell spreading. Data are presented as average cell area ± SEM from a minimum of 100 cells for each condition. ^*∗*^*p* < 0.001, ^*∗∗*^*p* < 0.005, and ^#^*p* < 0.05 indicate statistical significance relative to IgG1 Fc control; two-sample *t*-test.

Cell spreading requires integrins to form cell-substratum adhesions that link the ECM to the actin cytoskeleton in order to regulate cell size and shape and initiate cell migration [10, 27–29]. Similar to motility, cells have been shown to exhibit a biphasic spreading response to increasing surface densities of collagen [9]. Given the observation that Sema3A increases integrin expression in breast carcinoma cells [20] as well as our observations regarding the effects of Sema3A on cell motility on different substrate concentrations, we sought to determine whether Sema3A alters the spreading response of MDA-MB-231 cells to increasing coating concentrations of various ECM. Cell spreading was assessed by measuring cell area following attachment to substrata coated with different concentrations of ECM in the presence or absence of Sema3A. On all tested substrata, there was a positive correlation between substratum coating concentration and cell area (Figure 3). These data are consistent with previous studies showing a similar relationship between ECM coating density and cell spreading [10, 27, 28]. At substrata coated with lower (0.1–10 *μ*g/mL) collagen concentrations, Sema3A increased cell area but inhibited cell spreading at high coating concentrations of collagen (Figures 3(a) and 3(b)). For instance, at substrata coated with 1.0 ug/mL collagen, Sema3A increased cell area compared to IgG1 Fc control (average cell area = 816.7 ± 357.82 um^2^ for IgG1 Fc control versus 1013.8 ± 363.52 um^2^ for Sema3A). However, at substrata coated with high concentrations of collagen, Sema3A inhibited cell spreading (average cell area = 1088.7 ± 363.2 um^2^ for IgG1 Fc control versus 957.0 ± 324.0 um^2^ for Sema3A). Similar, but less robust, effects of Sema3A treatment were observed on fibronectin (Figure 3(c)). Sema3A inhibited cell spreading on substrata coated at intermediate and high concentrations of laminin but did not show any stimulatory effects on laminin (Figure 3(d)). Consistent with our observations on cell motility, Sema3A enhances cell spreading on low and intermediate substratum concentrations of collagen and fibronectin, but not laminin, while inhibiting spreading at high substratum coating concentrations.

**Figure 4:**
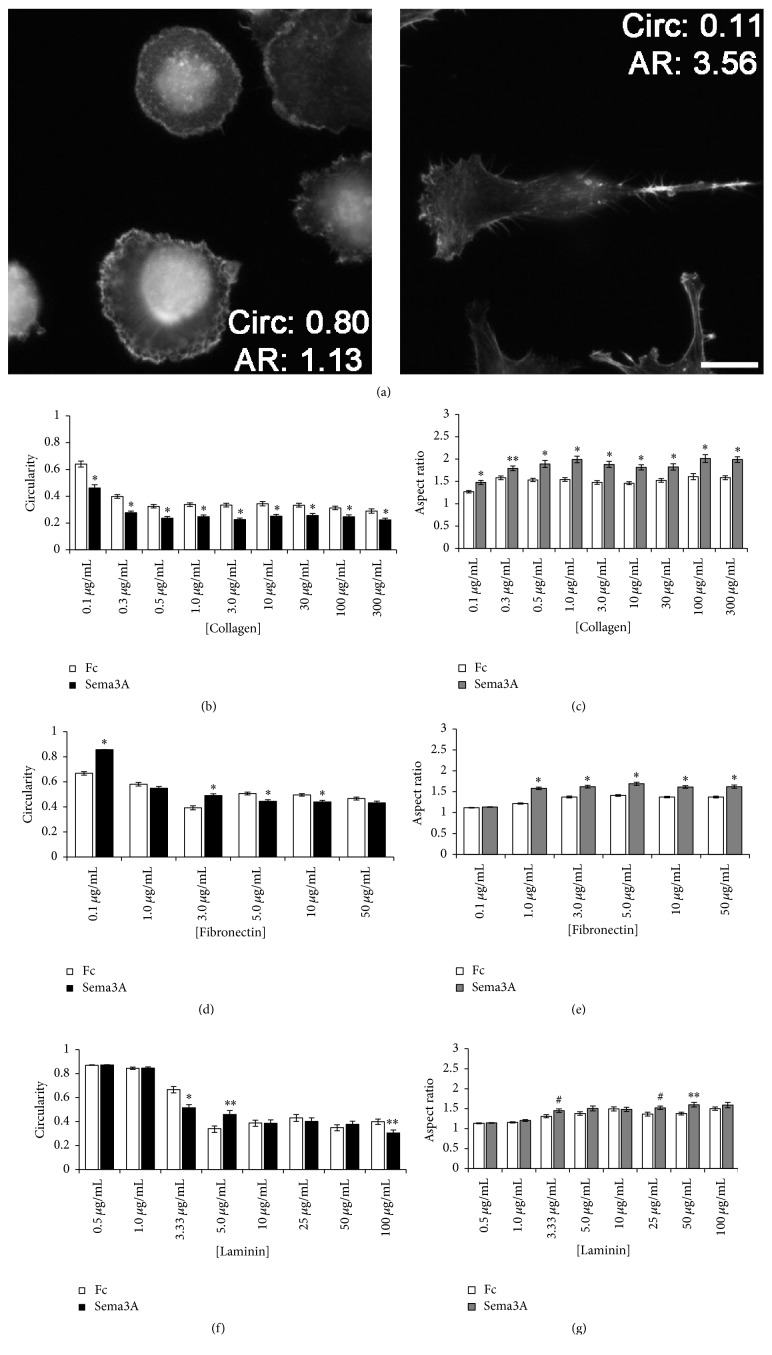
Sema3A alters cell morphology on collagen and fibronectin but has minimal effects on laminin. (a) Example images of cells stained with TRITC-phalloidin. Rounded cells display circularity close to 1 and a low aspect ratio (close to 1), while more elongated cells have circularity close to 0 and a high aspect ratio (greater than 1). Scale bar = 20 *μ*m. Cell circularity and aspect ratio were measured from cells exposed to substrata coated with increasing concentrations of collagen ((b) and (c)), fibronectin ((d) and (e)), and laminin ((f) and (g)). Sema3A consistently reduced circularity and increased aspect ratio on substrata coated with increasing concentrations of collagen, while the effects of Sema3A on fibronectin and laminin were less consistent. Graphed data represent the mean ± SEM from at least 80 cells for each condition. ^*∗*^*p* < 0.001, ^*∗∗*^*p* < 0.005, and ^#^*p* < 0.05 indicate statistical difference relative to IgG1 Fc control; two-sample *t*-test.

In addition to measuring cell area as an indication of integrin-based cell spreading, we assessed changes in cell morphology using both circularity and aspect ratio measurements. Cell morphology, in addition to motility and spreading, is regulated by integrin-mediated changes in cell-substratum adhesions that link the ECM to the actin cytoskeleton [10, 28, 30]. Cell circularity and aspect ratio both measure the roundness of a cell using different measured parameters. Cells with circularity close to 1 and a low aspect ratio (close to 1) are more circular, while cells with circularity close to 0 and a high aspect ratio (greater than 1) are more elongated (Figure 4(a)). Cells treated with Sema3A were more elongated on all coating concentrations of collagen as indicated by a decrease in circularity and increase in aspect ratio (Figures 4(b) and 4(c)). Although effects on circularity were more variable, increased aspect ratio suggested a more elongated morphology of cells treated with Sema3A on fibronectin (Figures 4(d) and 4(e)). Sema3A did not have any consistent effect on cell morphology on laminin as both circularity and aspect ratio measurements were variable (Figures 4(f) and 4(g)). For instance, on substrata coated with 5.0 *μ*g/mL laminin, Sema3A increased circularity, but Sema3A decreased circularity on substrata coated with 100 *μ*g/mL laminin. Interestingly, increasing coating concentrations of all tested ECM molecules promoted a more elongated morphology (Figures 4(b)–4(g)). These observations are consistent with previous studies demonstrating a density-dependent effect of ECM on cell morphology [31]. Overall, these results suggest that Sema3A promotes a more elongated morphology on collagen and fibronectin, while the effects of Sema3A on laminin are more variable. Collectively, the Sema3A-induced changes in MDA-MB-231 cell spreading and morphology, as well as cell motility, suggest that Sema3A may regulate changes in cell-substratum adhesions.

### 3.3. Sema3A Enhances pFAK^397^ at Focal Adhesions in a Substrate-Specific Manner

**Figure 5:**
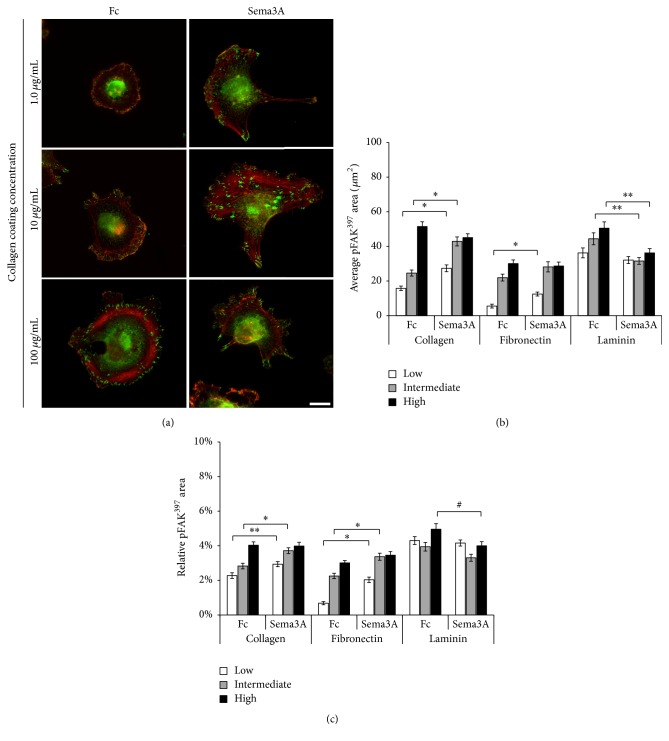
Sema3A increases pFAK^397^ levels at focal adhesions in cells plated on collagen and fibronectin, but not laminin. (a) Representative images of cells grown on substrata coated with low, intermediate, and high concentrations of collagen. Indirect immunofluorescence of focal adhesions using an antibody for phosphorylated FAK^397^ (green) and counterstained with phalloidin (red). Scale bar = 10 *μ*m. (b) Sema3A increased the average total surface area of pFAK^397^ by ~74% on substrata coated with low and intermediate concentrations of collagen, while Sema3A produced an increase of 127% on substrata coated with low concentrations of fibronectin. pFAK^397^ staining was not statistically different at high coating concentrations of collagen or fibronectin. Interestingly, Sema3A reduced the average total surface area of pFAK^397^ by 11–29% on all coating concentrations of laminin. (c) Relative pFAK^397^ area as analyzed by the average total surface area occupied by pFAK^397^ normalized to total cell area as determined by TRITC-phalloidin. Data are presented as average ± SEM from a minimum of 50 cells for each condition. ^*∗*^*p* < 0.001, ^*∗∗*^*p* < 0.005, and ^#^*p* < 0.05 indicate statistical significance relative to IgG1 Fc control; two-sample *t*-test.

Cells attach to the ECM through integrin-mediated adhesions that link the ECM to the actin cytoskeleton in order to regulate various cellular responses such as cell migration and metastasis, adhesion, and proliferation [3]. Upon integrin engagement with the ECM, various adaptor proteins and intracellular signaling molecules are recruited to the cytoplasmic domain of integrin receptors to serve as a scaffold between the ECM and the actin cytoskeleton [29]. For instance, integrin binding to the ECM induces focal adhesion kinase (FAK) to become phosphorylated at tyrosine 397 (pFAK^397^) which activates other downstream targets to regulate focal adhesions and other cellular responses [32]. Furthermore, FAK is phosphorylated and localized at focal adhesions upon cell adhesion to the ECM [32]. Therefore, we assessed focal adhesions using a pFAK^397^ antibody to measure the effects of Sema3A on cells plated on substrata coated with increasing concentrations of collagen, fibronectin, and laminin. Increasing coating concentrations of all tested substrata produced an increase in the average area of pFAK^397^-containing focal adhesions (Figures 5(a) and 5(b)). Furthermore, Sema3A enhanced the average pFAK^397^ area on substrata coated with low and intermediate concentrations of collagen but produced no further effect on high collagen coating concentrations (Figure 5(b)). The effects of Sema3A on fibronectin were not as robust, as Sema3A enhanced the average pFAK^397^ area on substrata coated with low concentrations of fibronectin but had no significant effect at intermediate and high coating concentrations of fibronectin. Interestingly, Sema3A reduced the average pFAK^397^ area on substrata coated with intermediate and high concentrations of laminin. To account for the effects of Sema3A on increased cell spreading (Figure 3), relative pFAK^397^ area was calculated by normalizing the average pFAK^397^ area to the average cell area (measured using TRITC-phalloidin) for each condition. Similar trends were observed for relative pFAK^397^ area on all three substrata as was observed for average pFAK^397^ area (Figure 5(c)). Furthermore, similar results were obtained when focal adhesions were measured using a vinculin antibody (data to shown). These results indicate that Sema3A enhances MDA-MB-231 cell migration and spreading on nonoptimal coating concentrations of collagen and fibronectin through increased pFAK^397^ levels at focal adhesions.

### 3.4. Sema3A-Mediated Changes in MDA-MB-231 Cell Migration, Spreading, and pFAK^397^ Levels at Focal Adhesions Involve ROCK Signaling

**Figure 6:**
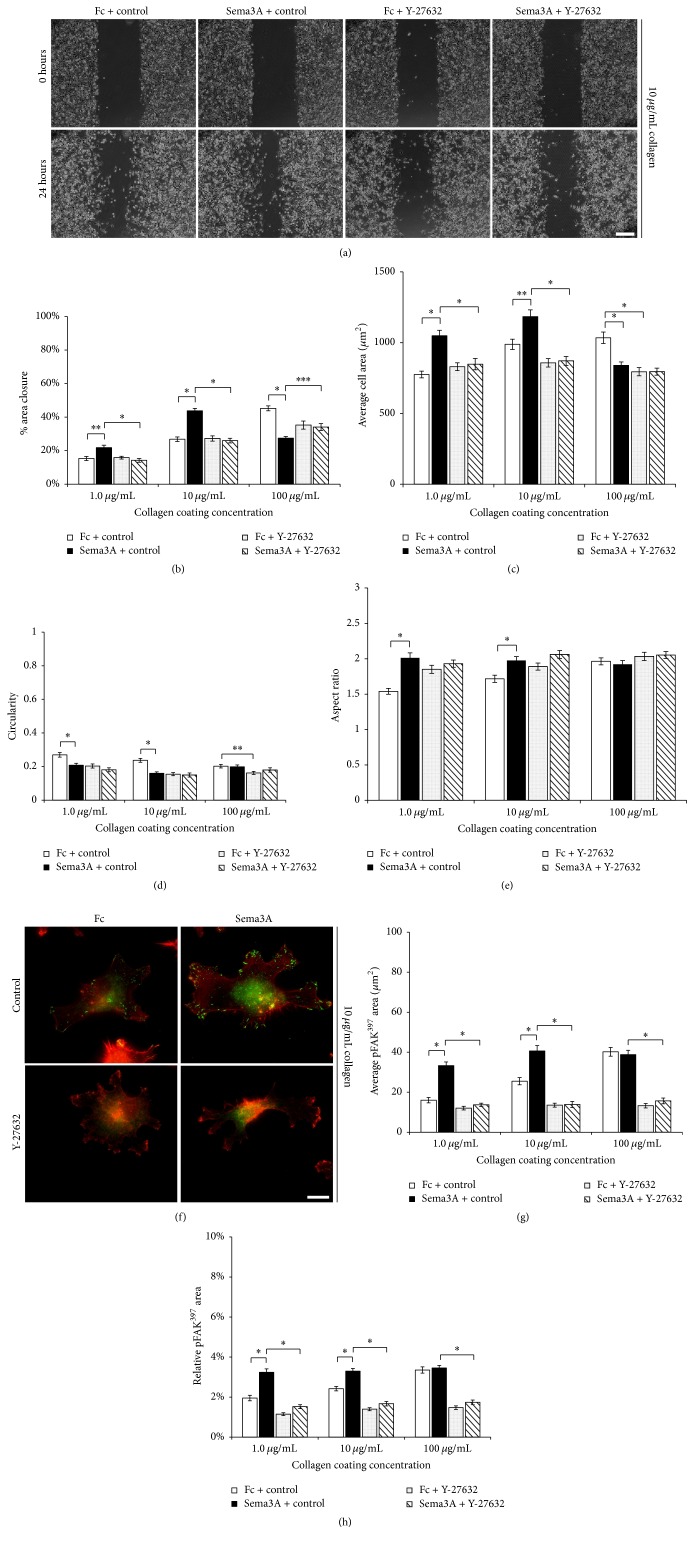
Inhibition of ROCK blocks the Sema3A-mediated effects on cell migration, spreading, and pFAK^397^ levels at focal adhesions. (a) Representative phase-contrast images from a scratch assay of cells cultured on substrata coated with 10 *μ*g/mL collagen in the presence of 100 *μ*g/mL IgG1 Fc control or Sema3A with and without 10 *μ*M Y-27632. Scale bar = 100 *μ*m. (b) Y-27632 blocked the Sema3A-mediated effects on all coating concentrations of collagen as measured using % area closure. Data are presented as average % area closure ± SEM from four independent experiments. (c) Average cell area of cells cultured on substrata coated with low, intermediate, and high concentrations of collagen. Cells were treated with IgG1 Fc control or Sema3A in the absence or presence of Y-27632. Y-27632 blocked the effects of Sema3A on all coating concentrations of collagen. Graphed data represent the mean ± SEM from at least 120 cells for each condition. ((d) and (e)) The effects of Y-27632 on control- and Sema3A-treated cells were assessed using cell circularity (d) and aspect ratio (e). While Sema3A alone decreased circularity and increased aspect ratio on substrata coated with low and intermediate concentrations of collagen, Y-27632 alone produced a similar effect. Data are presented as mean ± SEM from a minimum of 120 cells for each condition. (f) Y-27632 inhibited pFAK^397^ staining levels (green) at focal adhesions in both IgG1 Fc control- and Sema3A-treated conditions. Cells were counterstained using TRITC-phalloidin (red). Scale bar = 10 *μ*m. ((g) and (h)) The average total surface area of pFAK^397^ (g) and relative pFAK^397^ normalized to total cell area (h) for control- and Sema3A-treated cells exposed to substrata coated with low, intermediate, and high concentrations of collagen in the presence or absence of Y-27632. Graphed data represent the mean ± SEM from at least 50 cells for each condition. ^*∗*^*p* < 0.001, ^*∗∗*^*p* < 0.005, and ^*∗∗∗*^*p* < 0.01 indicate statistical significance relative to IgG1 Fc control for all graphed data; two-sample *t*-test.

The Rho family of GTPases are important regulators of focal adhesions [33–35]. For instance, RhoA stabilizes focal adhesions through Rho kinase- (ROCK-) dependent activation of myosin II [29, 36]. Furthermore, integrin-mediated adhesion to the ECM regulates the activity of Rho GTPases, which alter myosin-induced contractility and actin cytoskeletal dynamics [29]. Sema3A signaling alters cytoskeletal dynamics through changes in Rho GTPase signaling [37]. Although both integrin and Sema3A signaling pathways regulate RhoA activity, it is not clear whether Sema3A requires a RhoA-dependent mechanism to alter focal adhesions to regulate cell motility and spreading in response to changes in the coating concentrations of ECM. As a result, we assessed the effects of an inhibitor to the RhoA effector, ROCK, on Sema3A-induced changes in cell motility, spreading, and focal adhesions. Since Sema3A produced the most dramatic effects on cell migration, cell spreading, and pFAK^397^ levels at focal adhesions on collagen, we tested the effects of the ROCK inhibitor, Y-27632, on changes in cellular responses to increasing coating concentrations of collagen. On substrata coated with low and intermediate concentrations of collagen, Y-27632 completely blocked the Sema3A-enhanced changes in area closure (Figures 6(a) and 6(b)). However, on substrata coated with high concentrations of collagen, Y-27632 partially disrupted the Sema3A-induced inhibition on cell migration, suggesting that Sema3A may utilize additional signaling pathways as collagen coating concentration increases. Y-27632 completely blocked the Sema3A-induced increases in cell area on low and intermediate coating concentrations of collagen (Figure 6(c)). However, on substrata coated with high concentrations of collagen, Y-27632 alone produced similar effects as Sema3A alone on cell area. But, Y-27632 had no additional effect on cell area when combined with Sema3A. Given the observation that the effects of Sema3A and Y-27632 were not additive suggests Sema3A signals through ROCK to regulate cell spreading. Interestingly, Y-27632 alone produced changes in cell morphology that were similar to Sema3A alone (Figures 6(d) and 6(e)). However, Y-27632 produced no additional changes in cell morphology when combined with Sema3A. Finally, Y-27632 blocked Sema3A-induced increases in average pFAK^397^ area and relative pFAK^397^ area on all concentrations of collagen (Figures 6(f)–6(h)). Together, these data suggest that Sema3A may require a ROCK-dependent mechanism to mediate its effects on MDA-MB-231 cell migration and spreading, perhaps by regulating pFAK^397^ levels at focal adhesions.

## 4. Discussion

The extracellular matrix, as well as other extracellular ligands, plays a critical role in tumor cell invasion and metastasis. However, the means by which cells integrate numerous extracellular cues to induce changes in cellular responses are not well understood. This study demonstrates that Sema3A is an important regulator of MDA-MB-231 cell migration, spreading, and FAK signaling at focal adhesions in response to increasing substrate concentrations of ECM. First, we show that Sema3A shifts the response curve for MDA-MB-231 cell motility and spreading such that Sema3A enhances cell migration and spreading on substrata coated with low and intermediate concentrations of collagen and fibronectin, but not laminin. However, on substrata coated with high concentrations of collagen and fibronectin, Sema3A inhibits cell migration and spreading. To our knowledge, this is the first time Sema3A has been documented to have both stimulatory and inhibitory effects on cell motility and spreading in response to changes in substrate concentration. In addition, Sema3A alters cell shape on all coating concentrations of collagen and fibronectin but does not have as substantial an effect on laminin. Second, Sema3A enhances pFAK^397^ at focal adhesions on collagen and fibronectin but reduces pFAK^397^ levels at focal adhesions on laminin. Finally, preliminary results suggest that inhibition of ROCK blocks the Sema3A-induced changes in cell migration, cell spreading, and pFAK^397^ levels at focal adhesions in response to increasing substrate concentrations of collagen. These results support a role for Sema3A in regulating cellular responses to changes in substratum concentrations by modulating pFAK^397^ levels at focal adhesions which may include a ROCK-dependent pathway.

The strength of cell-substratum adhesions is influenced by the concentration of the substratum, the level of integrin expression, integrin-ligand affinity, or integrin-cytoskeletal interactions [5–8]. The degree of cell migration and spreading is influenced by the strength of cell-substratum adhesions where an optimum substrate concentration supports maximal migration and spreading [5–9]. Our observation that increasing substratum concentration enhanced migration speed and spreading (Figures 1–3) is consistent with published studies. Interestingly, Sema3A treatment shifted the maximal motility and spreading response of MDA-MB-231 cells to lower coating concentrations of collagen and fibronectin, but not laminin, while inhibiting cell motility and spreading at high substrate concentrations (Figures 1–3). Furthermore, our results suggest the Sema3A-mediated motility and spreading response may be a result of increased pFAK^397^ levels at focal adhesions (Figure 5). Indeed, Sema3A treatment of cells on collagen produced the greatest shift in the motility and spreading response (Figures 1–4), while also producing the most robust effect on pFAK^397^ levels at focal adhesions (Figure 5). Studies have shown that maximal cell migration is governed by a reciprocal relationship between substrate concentration and integrin expression [6, 8]. For instance, decreased integrin-ligand affinity enhances cell migration on high substrate concentration, while increased integrin-ligand affinity promotes maximal cell migration at lower concentrations of substratum [6, 8]. Furthermore, disrupted integrin-cytoskeletal linkages perturbed the migration and spreading of CHO cells, providing additional evidence that the strength of the cell-substratum adhesions is important to regulate cellular responses to changes in substratum concentration [8]. While studies have demonstrated that Sema3A upregulates *α*2*β*1 integrin expression in breast tumor cells when plated on collagen [20], a future direction for this research would be to measure integrin expression levels on cells plated on different concentrations of ECM in order to fully understand the relationship between cell-substratum adhesion strength and cell migration and spreading in response to Sema3A. In light of this, our findings suggest that Sema3A tunes the cellular responses to nonoptimal ECM coating concentrations through increased focal adhesions.

Sema3A has been shown to increase integrin expression and cell adhesion in breast carcinoma cells [20], while our results demonstrate that Sema3A increases integrin signaling, as indicated by increased pFAK^397^ at focal adhesions, on collagen and fibronectin (Figure 5). However, the mechanisms by which Sema3A regulates integrin activation or integrin-mediated signaling are not well understood. Studies suggest Sema3A mediates its effects through integrin-mediated FAK phosphorylation. For instance, Sema3A regulates axon remodeling of mouse hippocampal neurons through a FAK-dependent pathway [22]. Furthermore, inactivation of *β*1 integrin or FAK blocked the Sema3A-induced increases in dendrite extension of hippocampal neurons [21]. Our results suggest that Sema3A-mediated changes in cell migration, spreading, and pFAK^397^ at focal adhesions may require ROCK signaling (Figure 6). Our findings are consistent with other studies that showed Sema3A treatment of breast carcinoma cells increases RhoA activity to inhibit cell migration [38]. However, our results suggesting that Sema3A may require ROCK signaling to modulate MDA-MB-231 cell migration, spreading, and pFAK^397^ levels at focal adhesions are preliminary. With that said, whether Sema3A regulates integrin signaling through a FAK-dependent pathway, or some other signaling pathway that modulates RhoA activity, in response to increasing substratum concentrations remains a subject for future investigation.

Our observations suggest that Sema3A stimulates changes in MDA-MB-231 breast carcinoma cell motility, spreading, and focal adhesions in a substrate-specific manner. Sema3A appears to have the most robust effects on cells plated on collagen or fibronectin, while the response of cells plated on laminin was minimal. These observations imply that Sema3A may modulate the cellular response in an integrin-specific manner in breast carcinoma cells. Indeed, type I collagen or fibronectin inhibited paclitaxel-induced apoptosis in MDA-MB-231 breast epithelial cells, while laminin-1 had no protective effect [39]. Furthermore, the protective effects of collagen I and fibronectin were mediated through the *α*2*β*1 and *α*5*β*1 integrins, respectively. These findings suggest that breast carcinoma cells may respond differently to Sema3A based on different expression profiles of integrins. Further investigation is needed to assess the effects of Sema3A on the expression of different integrin subunits that might confer a selective response of Sema3A to different types of ECM.

Sema3A can have both antitumorigenic and protumorigenic effects on cells. For instance, Sema3A inhibits tumor growth, migration, and metastasis of breast and prostate cancer cells as well as melanoma [14–17] but facilitates pancreatic and colon cancer cell invasion [40, 41]. Furthermore, the effects of Sema3A on integrin activation and cell adhesion appear to be cell-type specific. Sema3A inhibits cell adhesion in prostate cancer cells as well as dorsal root ganglion neurons [14, 24] but promotes integrin activation and cell adhesion in breast cancer cells [20]. One explanation for the cell-type specific responses to Sema3A may be due to the different binding states of the Sema3A coreceptors, plexin A1 and neuropilin-1. For example, perturbation of L1 CAM, which directly interacts with neuropilin-1, has been shown to switch Sema3A-induced neuronal growth cone repulsion into attraction [42]. In fact, numerous extracellular guidance cues have been shown to be either attractive or repulsive towards neuronal growth cones depending on cellular context [43]. For instance, cultured rat cerebellar axons turned away from a source of stromal-derived factor 1 (SDF-1) through G protein-coupled receptor activation of protein kinase C (PKC), while inhibition of PKC converted the SDF-1-induced axon repulsion into attractive turning towards the source of SDF-1 [44]. In light of the findings of this study, it is feasible that Sema3A may alter FAK phosphorylation at focal adhesions to regulate the cellular responses to changes in ECM composition of the stroma during tumor progression. However, the mechanisms by which Sema3A signaling modulates integrin function, or vice versa, to switch the motility and spreading response of breast epithelial cells when plated on different concentrations of ECM remain a subject for future investigation.

The observations from this study may have both physiological and pathological implications as they may explain some of the underlying mechanisms by which changes in ECM composition during normal tissue morphogenesis as well as pathogenesis may contribute to tumor progression and metastasis. Invasive carcinomas undergo alterations in the expression levels of integrins as well as changes in deposition of stromal ECM [2, 45]. For instance, *α*2*β*1 integrin (which binds mainly collagen) and other integrin subunits, including *α*1, *α*6, *β*1, or *β*4 integrin subunits, are downregulated in breast epithelial neoplasms [46]. Collagens (I, III, and V) and fibronectin, among other ECM constituents, are upregulated in the stroma of invasive mammary carcinomas [45, 47]. In addition, Sema3A is considered to function as a tumor suppressor as reduced Sema3A expression is correlated with breast carcinoma and melanoma progression in humans [17, 19], while overexpression of Sema3A suppresses tumor growth and metastasis in vivo using xenograft mouse models [16–18]. In fact, studies have shown that breast carcinoma cells secrete Sema3A which signals through an autocrine mechanism to upregulate *α*2*β*1 integrin expression [26]. This study provides evidence implicating a role for Sema3A signaling in modulating cell-substratum adhesions as integrin expression, ECM deposition, and Sema3A expression are altered during mammary tumor progression. However, future investigations should focus on identifying and characterizing the mechanisms of how breast carcinoma cells integrate numerous extracellular cues through integrin and nonintegrin receptors to regulate cell-substratum interactions during tumor cell invasion and metastasis.

## Supplementary Material

Supplemental Figure 1. Sema3A has no significant effect on cell proliferation. Cells were treated with IgG1 Fc control or Sema3A for 24 hours on substrata coated with different concentrations of collagen (A), fibronectin (B), or laminin (C). Cell proliferation was determined using CellTiter 96 AQ_ueous_ One Solution Reagent and absorbance was measured at 490nm. Data are presented as average absorbance ± SEM from eight wells.

## Abbreviations

Sema3A: Semaphorin 3A
ECM: Extracellular matrix
FAK: Focal adhesion kinase
ROCK: Rho-associated protein kinase.
