# Supplementary material for: Semaphorin 3A Increases FAK Phosphorylation at Focal Adhesions to Modulate MDA-MB-231 Cell Migration and Spreading on Different Substratum Concentrations
Source: Int J Breast Cancer. 2017 Jan 15;2017:9619734. doi: 10.1155/2017/9619734 (PMC5274681; doi:10.1155/2017/9619734)
Supplement: Supplementary file 1 — Supplemental Figure 1. Sema3A has no significant effect on cell proliferation. Cells were treated with IgG1 Fc control or Sema3A for 24 hours on substrata coated with different concentrations of collagen (A), fibronectin (B), or laminin (C). Cell proliferation was determined using CellTiter 96 AQueous One Solution Reagent and absorbance was measured at 490nm. Data are presented as average absorbance ± SEM from eight wells. [file 9619734.f1.pdf]

Supplemental Figure 1

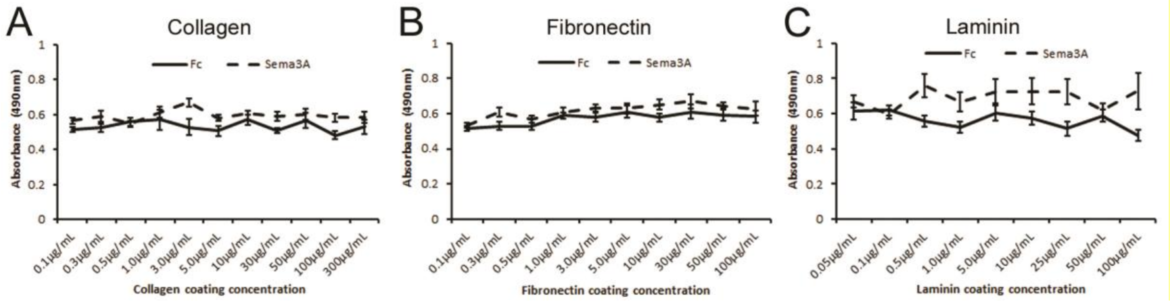

Supplemental Figure 1. Sema3A has no significant effect on cell proliferation. Cells were treated with IgG1 Fc control or Sema3A for 24 hours on substrata coated with different concentrations of collagen (A), fibronectin (B), or laminin (C). Cell proliferation was determined using CellTiter 96® AQ_{ueous} One Solution Reagent and absorbance was measured at 490nm. Data are presented as average absorbance ± SEM from eight wells.
